# Burn Eschar Stimulates Fibroblast and Adipose Mesenchymal Stromal Cell Proliferation and Migration but Inhibits Endothelial Cell Sprouting

**DOI:** 10.3390/ijms18081790

**Published:** 2017-08-18

**Authors:** Hanneke N. Monsuur, Lenie J. van den Broek, Renushka L. Jhingoerie, Adrianus F. P. M. Vloemans, Susan Gibbs

**Affiliations:** 1 Department of Dermatology, VU University Medical Center, Amsterdam Movement Sciences, 1081 HZ Amsterdam, The Netherlands; h.monsuur@vumc.nl (H.N.M.); l.vandenbroek@vumc.nl (L.J.v.d.B.); renushkajhingoerie@gmail.com (R.L.J.); 2 Burn Center, Red Cross Hospital, 1942 LE Beverwijk, The Netherlands; jvloemans@rkz.nl; 3 Department of Oral Cell Biology, Academic Center for Dentistry Amsterdam (ACTA), University of Amsterdam and VU University Amsterdam, Amsterdam Movement Sciences, 1081 HZ Amsterdam, The Netherlands

**Keywords:** burn, wound healing, wound extract, granulation tissue, endothelial cell, fibroblast, ASC, skin

## Abstract

The majority of full-thickness burn wounds heal with hypertrophic scar formation. Burn eschar most probably influences early burn wound healing, since granulation tissue only forms after escharotomy. In order to investigate the effect of burn eschar on delayed granulation tissue formation, burn wound extract (BWE) was isolated from the interface between non-viable eschar and viable tissue. The influence of BWE on the activity of endothelial cells derived from dermis and adipose tissue, dermal fibroblasts and adipose tissue-derived mesenchymal stromal cells (ASC) was determined. It was found that BWE stimulated endothelial cell inflammatory cytokine (CXCL8, IL-6 and CCL2) secretion and migration. However, BWE had no effect on endothelial cell proliferation or angiogenic sprouting. Indeed, BWE inhibited basic Fibroblast Growth Factor (bFGF) induced endothelial cell proliferation and sprouting. In contrast, BWE stimulated fibroblast and ASC proliferation and migration. No difference was observed between cells isolated from dermis or adipose tissue. The inhibitory effect of BWE on bFGF-induced endothelial proliferation and sprouting would explain why excessive granulation tissue formation is prevented in full-thickness burn wounds as long as the eschar is still present. Identifying the eschar factors responsible for this might give indications for therapeutic targets aimed at reducing hypertrophic scar formation which is initiated by excessive granulation tissue formation once eschar is removed.

## 1. Introduction

One of the most frequent causes of full-thickness burn wounds is exposure to hot water or (flash) fire. A full-thickness burn wound results in loss of viable epidermis and dermis. Currently, small full-thickness burns (<15% Total Body Surface Area; TBSA) are treated conservatively for 10–14 days, followed by debridement of eschar and application of a split skin autograft to deeper, non healing regions. To prevent a Severe Systemic Inflammation Syndrom (SIRS), burns larger than 15% TBSA require earlier excision followed by debridement and application of a split skin autograft [1,2]. The majority of full-thickness burn wounds result in the formation of hypertrophic scars, independent of the treatment strategy [3]. In order to develop improved treatment strategies to prevent hypertrophic scar formation, a better understanding of the early stages of wound healing and the influence of eschar on the early healing process is required.

Wound healing of full-thickness burns differs from normal wound healing in several aspects, notably alterations in haemostasis, inflammation and granulation tissue formation [4]. Burn injury coagulates the superficial blood vessels hindering fibrin clot formation, and when the wound is debrided excessive bleeding is triggered, leading to haemostasis, followed by the formation of granulation tissue within a few days [4]. Clinical observations show that the presence of eschar on the wound bed prohibits granulation tissue formation. Granulation tissue is characterized by a high density of fibroblasts, granulocytes, macrophages and microcapillaries. In full-thickness burn wounds the cells required for wound healing have to migrate from the wound edges, from the subcutaneous adipose tissue or other origins. In addition to dermal fibroblasts and dermal-endothelial cells (dermal-EC) migrating from the wound edges also adipose tissue-derived mesenchymal stromal cells (ASC) and adipose-endothelial cells (adipose-EC) are likely to be involved and may contribute to less favorable wound healing and hypertrophic scar formation. For example, the persistent myofibroblasts in hypertrophic scars may originate from the adipose tissue, since a high percentage of ASC express the myofibroblast marker α-smooth muscle actin [5,6,7]. Also a possible contribution of endothelial cells to hypertrophic scar formation has been suggested as hypertrophic scars contain more microcapillaries than normal scars [8,9]. The alterations in haemostasis, granulation tissue formation and the contribution to wound healing by cells from alternative origins, such as adipose tissue can contribute to the increased risk of hypertrophic scar formation as seen in many full-thickness burn wounds [4].

Since granulation tissue only forms after the burn wound eschar has been removed, burn eschar is most likely to strongly influence early healing of the burn wound. To investigate this further, burn wound extract (BWE) can be isolated from the interface between non-viable eschar and viable tissue and used to represent the burn wound environment, allowing us to study the cellular and molecular components involved in burn wound healing [10,11]. Previously we have shown that this BWE is highly bioactive containing abundant levels of many cytokines, chemokines and growth factors such as CCL2, CCL5, CCL18, CCL20, CCL27, IL-1α, IL-6, CXCL1, CXCL8, basic fibroblast growth Factor (bFGF), hepatocyte growth factor and transforming growth factor-β. BWE could further stimulate ASC and fibroblasts to secrete more mediators related to inflammation, angiogenesis and granulation tissue formation resulting in an amplified inflammatory response [11].

In this study, to further investigate the effect of eschar-derived BWE on delayed granulation tissue formation we focused on endothelial cells derived from the dermis and adipose tissue. The influence of BWE on endothelial cell inflammation, migration, proliferation and angiogenic sprouting was determined. Our results indicate that BWE from full-thickness burn wounds stimulates the secretion of inflammatory proteins and endothelial cell migration, but inhibits endothelial cell proliferation and vessel sprouting. In contrast to the findings with dermal- and adipose-EC, stimulation of both proliferation and migration was seen with fibroblasts and ASC in the presence of BWE.

## 2. Results

### 2.1. Eschar

**Figure 1:**
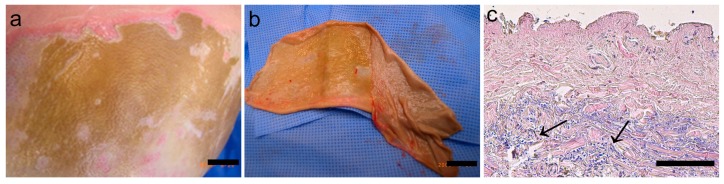
Macroscopic pictures and morphology of human eschar tissue. (**a**) Eschar tissue on patient; (**b**) Eschar after tangential excision; (**c**) Eschar at interface of non-viable and viable tissue from which acellular Burn Wound Extract (BWE) is derived (see Materials and Methods); hematoxylin and eosin staining showing the absence of epidermis; small, rounded cells are present in the lower levels of the dermis (areas with blue nuclei; see arrows). Scale bar = 1 cm (**a**,**b**) or 200 µm (**c**).

Eschar was removed from the patient by (tangential) excision (Figure 1). Eschar at the interface between non-viable and viable tissue was used to obtain an acellular BWE for the experiments described in this study. Characteristics of the eschar and BWE are shown in Table 1. The histology of this eschar showed absence of an epidermis and a tissue containing many small, rounded cells in the lower eschar layers (Figure 1c). It has previously been reported that eschar contains viable cells resembling ASC [12].

### 2.2. Burn Wound Extract Inhibits Endothelial Cell Proliferation and Sprouting

Previously we have shown that eschar BWE contains a large reservoir of bioactive cytokines and chemokines [11]. In order to determine the effect of BWE on cells underneath the eschar, both dermal-EC and adipose-EC were exposed to BWE. The influence of BWE on (i) inflammatory cytokine secretion was determined by ELISA; (ii) cell migration was determined using the wound healing scratch assay and (iii) proliferation by 3H incorporation.

**Figure 2:**
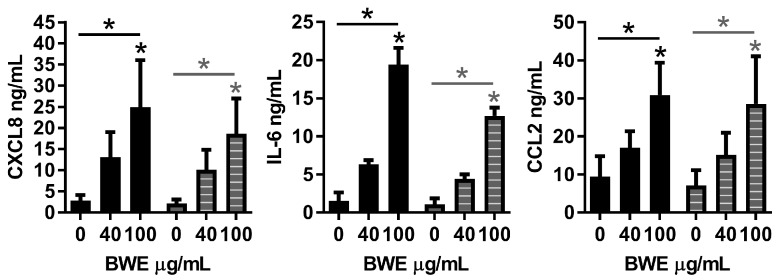
Secretion of inflammation factors by dermal- and adipose-endothelial cells. Secretion of CXCL8, IL-6 and CCL2 after a 24 h exposure to 0, 40 or 100 µg/mL BWE. Basal amounts of protein in culture medium containing 100 µg/mL BWE without cells: CXCL8: <2 ng/mL; IL-6: <1 ng/mL; CCL2: <0.5 ng/mL. Significance of the dose response curve was calculated using a one-way ANOVA followed by a Dunn’s multiple comparison test and significance of basic Fibroblast Growth Factor (bFGF) induction was tested with a *t*-test; * *p* < 0.05. Data is shown for 3 independent experiments as mean ± SEM. Each experiment represents a different cell donor and a different BWE donor (see Table 1). Black, solid bars represent dermal-endothelial cells (dermal-EC) and grey, striped bars adipose-endothelial cells (adipose-EC).

**Figure 3:**
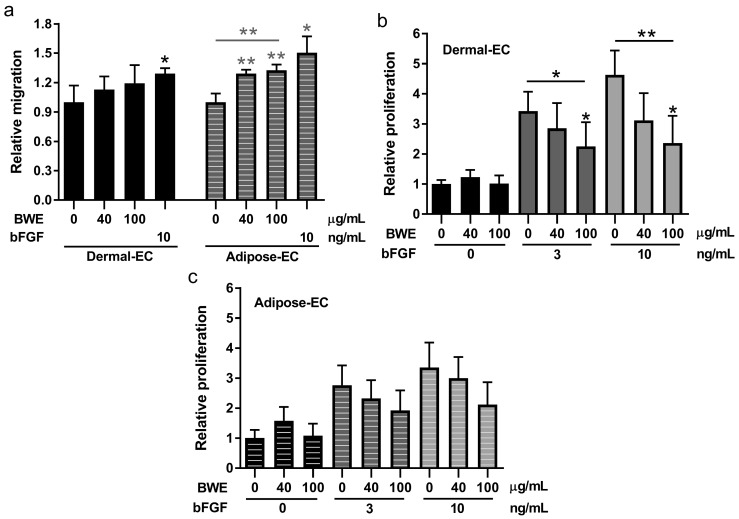
Migration scratch assay and proliferation assay using dermal- and adipose-endothelial cells. (**a**) Relative migration values of dermal- and adipose-EC cultured in the presence of 0, 40 or 100 µg/mL BWE or 10 ng/mL bFGF. Relative migration is calculated from the scratch area closed compared to unexposed endothelial cells; (**b**) Proliferation (3H incorporation) values, relative to unexposed endothelial cells, of dermal-EC cultured in the presence of 0, 3 or 10 ng/mL bFGF in combination with BWE; (**c**) Proliferation values, relative to unexposed endothelial cells, of adipose-EC cultured in the presence of 0, 3 or 10 ng/mL bFGF combined with BWE. Significance of the dose response curve was calculated using a one-way ANOVA followed by a Dunn’s multiple comparison test and significance of bFGF induction was tested with a *t*-test; * *p* < 0.05, ** *p* < 0.01. Data is shown for 4 independent experiments as mean ± SEM. Each experiment represents a different cell donor and a different BWE donor (see Table 1). Solid bars represent dermal-EC and striped bars adipose-EC.

BWE exposure increased CXCL8, IL-6 and CCL2 secretion by dermal- and adipose-EC in a dose dependent manner in line with our previous findings for fibroblasts and ASC (Figure 2). CXCL8 secretion was induced by 8.2 and 8.9-fold, IL-6 by 37.3 and 28.1-fold and CCL2 by 4.4 and 4.7-fold (dermal- and adipose-EC respectively; 100 µg/mL). Migration of adipose-EC was also stimulated during a time period of 16 h in the scratch wound-healing assay. For dermal-EC a relative increase of 1.19-fold (100 µg/mL) was observed compared to the bFGF positive control, which showed 1.29-fold increase (not significant) (Figure 3a). For adipose-EC a relative increase of 1.33-fold (100 µg/mL) was observed compared to the bFGF positive control, which showed 1.50-fold increase (*p* < 0.01) (Figure 3a). The morphology of the cells was not affected by the addition of BWE (Supplementary Figure S1a). In contrast to cell migration, BWE did not influence the basal level of proliferation of endothelial cells (Figure 3b,c). Endothelial cell proliferation was stimulated by the addition of bFGF, for dermal-EC a relative increase of 4.63-fold was achieved by 10 ng/mL bFGF and for adipose-EC a relative increase of 3.35-fold (Figure 3b,c). Notably, when BWE was added in combination with bFGF, the bFGF stimulated increase in proliferation was inhibited in a dose-dependent manner (Figure 3b,c). The inhibitory effect was more pronounced for dermal-EC than for adipose-EC. The relative proliferation for dermal-EC was reduced by 49% and for adipose-EC by 37% when 10 ng/mL bFGF was combined with 100 µg/mL BWE.

**Figure 4:**
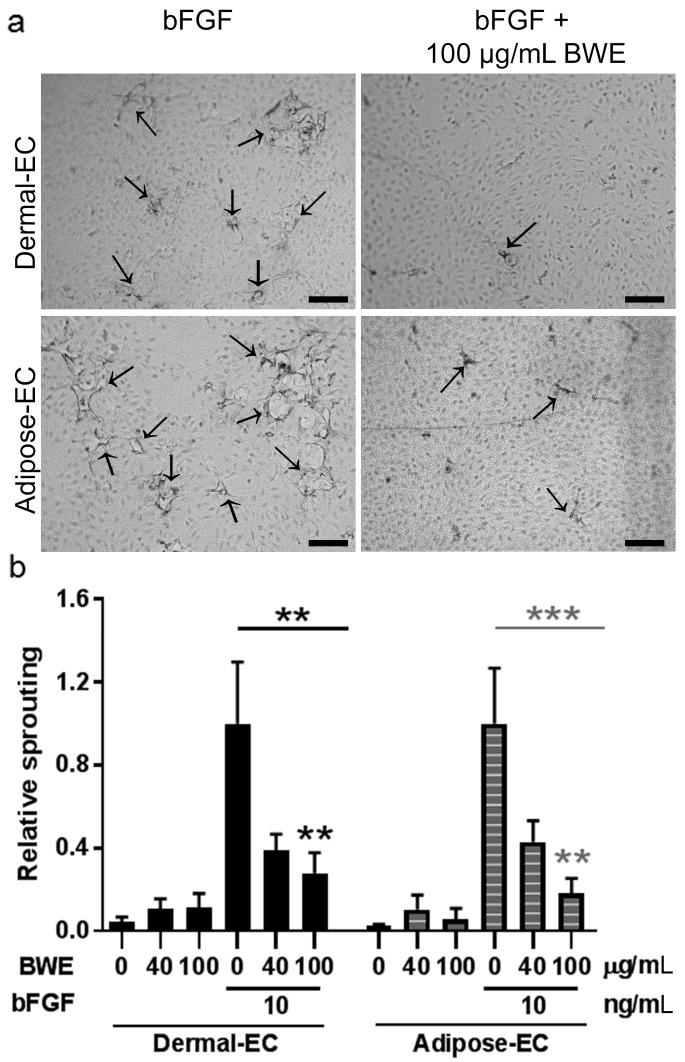
Sprouting assay using dermal- and adipose-endothelial cells. (**a**) Representative pictures of sprout formation of dermal- and adipose-EC into 3D fibrin matrices when exposed to 10 ng/mL bFGF or 10 ng/mL bFGF with 100 µg/mL BWE. Arrows indicate the sprouts; (**b**) Relative sprouting values of dermal- and adipose-EC in the presence of 0 or 10 ng/mL bFGF combined with 0, 40 or 100 µg/mL BWE compared to 10 ng/mL bFGF stimulated cultures. Significance of the dose response curve was calculated using a one-way ANOVA followed by a Dunn’s multiple comparison test; ** *p* < 0.01, *** *p* < 0.001. Data is shown for 6 independent experiments as mean ± SEM. Each experiment represents a different cell donor and a different BWE donor (see Table 1). Black, solid bars represent dermal-EC and grey, striped bars adipose-EC. Scale bars represent 50 µm.

Since angiogenesis involves a combination of cell proliferation, migration and matrix degradation we then determined the influence of BWE in a vessel sprouting assay. Sprout formation, as a measure for angiogenic response, was investigated using a 3D fibrin matrix. Endothelial cells seeded on top of this matrix will form sprouts into the matrix when an angiogenic stimulus is added to the medium [13]. Dermal- and adipose-EC did not form sprouts when exposed to BWE alone. When dermal- and adipose-EC were exposed to the angiogenic stimulus bFGF (10 ng/mL) induction of sprouting was clearly observed (Figure 4). Notably, this bFGF mediated increase in sprouting was inhibited by BWE in a dose-dependent manner. Dermal-EC showed 72% inhibition and adipose-EC showed 82% inhibition when 10 ng/mL bFGF was combined with 100 µg/mL BWE (Figure 4).

### 2.3. Burn Wound Extract Stimulates Both Migration and Proliferation of Fibroblasts and ASC

**Figure 5:**
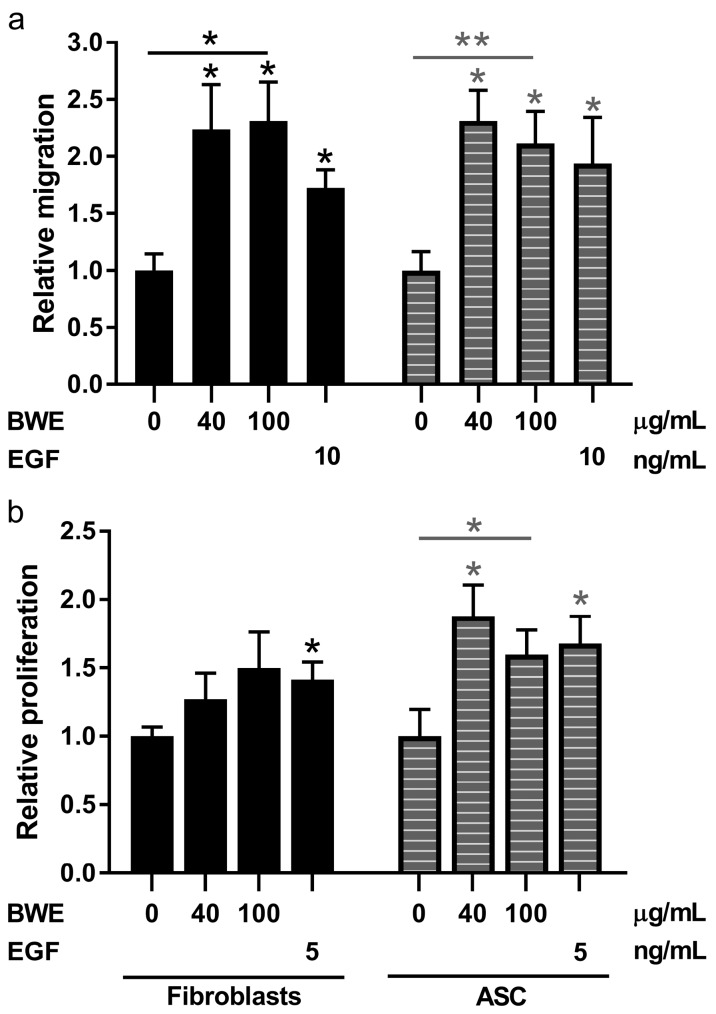
Migration scratch assay and proliferation assay using dermal fibroblasts and adipose tissue-derived mesenchymal stromal cells. (**a**) Relative migration values of fibroblasts and Adipose tissue-derived mesenchymal Stromal Cells (ASC) cultured in the presence of BWE or epidermal growth factor (EGF); (**b**) Relative proliferation values of fibroblasts and ASC cultured in the presence of BWE or EGF. The 5 and 10 ng/mL concentrations of EGF are optimal concentrations to serve as a positive control for the proliferation and migration experiments respectively. Significance of the dose response curve was calculated using a one-way ANOVA followed by a Dunn’s multiple comparison test and significance of EGF induction was tested with a *t*-test; * *p* < 0.05, ** *p* < 0.01. Data is shown for 4 independent experiments as mean ± SEM. Each experiment represents a different cell donor and a different BWE donor (see Table 1). Black, solid bars represent fibroblasts and grey, striped bars ASC.

Next the influence of BWE on fibroblast and ASC proliferation and migration was investigated. In contrast to endothelial cells, fibroblasts and ASC both showed a significant increase in migration in the scratch assay in the same order of magnitude as the epidermal growth factor (EGF) positive control (Figure 5a). For fibroblasts the highest relative increase of 2.31-fold was observed using 100 µg/mL BWE whereas EGF only showed 1.73-fold increase. For ASC the highest relative increase of 2.31-fold was observed using 40 µg/mL BWE whereas EGF only showed 1.94-fold increase. The morphology of the cells was not affected by the addition of BWE (Supplementary Figure S1b). BWE stimulated proliferation of fibroblasts and ASC, to the same extent as EGF (5 ng/mL) (Figure 5b). For fibroblasts the highest relative increase of 1.50-fold using 100 µg/mL BWE was observed whereas EGF showed 1.42-fold increase. For ASC the highest relative increase of 1.88 fold using 40 µg/mL BWE was observed whereas EGF showed 1.68 fold increase.

## 3. Discussion

The BWE derived from full-thickness burn wounds contains a very potent cocktail of bioactive cytokines, chemokines and growth factors representative of burn wound eschar [11]. In this study our focus was on the effect of BWE on endothelial cells from dermis and adipose tissue. BWE stimulated endothelial cells to secrete inflammatory proteins and enhanced endothelial cell migration. However, BWE had no effect on endothelial cell proliferation and angiogenic sprouting, and actually inhibited bFGF-mediated proliferation and sprouting. In contrast BWE stimulated both migration and proliferation of fibroblasts and ASC.

Our observation that BWE stimulated endothelial cells to secrete IL-6, CXCL8 and CCL2 was in agreement with our previous findings in which we showed that BWE stimulated inflammatory protein secretion by fibroblasts and ASC (but not keratinocytes) [11]. In the BWE there are many proteins present that can elicit an inflammatory response in endothelial cells, such as CCL2, IL-6, IL-1α, CXCL1 and CXCL8 [11]. No differences were found between dermal-EC and adipose-EC, however ASC were found to secrete more CCL2, IL-6 and CXCL8 in response to BWE than fibroblasts. The increased migration of endothelial cells, fibroblasts and ASC in the wound healing scratch assay may be attributed to the highly bioactive composition of the BWE that contains many chemotactic proteins such as bFGF and CCL5 [14,15]. Notably, we found that BWE inhibited endothelial cell proliferation and vessel sprouting. Vessel sprouting requires a combination of proliferation, migration and matrix breakdown [16,17]. Other studies investigating the effect of burn blister fluid on endothelial cells showed conflicting results, as endothelial cell proliferation, chemotaxis and angiogenesis were either stimulated or not affected [18,19,20]. However, blister fluid cannot be compared to our BWE, since blister fluid is obtained very early after injury (within 6–72 h) from (deep) partial-thickness burn wounds (compared to BWE which is isolated from full-thickness burn wounds between day 6 and 21). However, our results for fibroblasts and ASC were in line with results from blister fluid, which had a clear stimulatory effect on fibroblast proliferation and contraction [21,22].

In full-thickness burn wounds the eschar is often left on the burn wound for 10–14 days before a decision is made to remove the eschar by (tangential) excision. During this time period not only an alteration in wound healing is seen with regards to hemostasis, but also in granulation tissue formation. The removal of eschar causes excessive bleeding followed by hemostasis and (excessive) granulation tissue forms a few days after escharotomy [4]. An explanation for the inhibitory effect of BWE on bFGF-induced proliferation and sprouting may be the presence of inhibitory factors in the BWE, e.g., plasminogen activator inhibitor-1 or angiopoietin-2. The non-viable burned tissue might also release collagen-4 derived angiogenesis inhibitors into the BWE, for example arresten, canstatin or tumstatin [19]. Our findings give an explanation as to why excessive granulation tissue formation is prevented in full-thickness wounds as long as the eschar is still present. Further research is required to identify the factors present at the interface of non-viable eschar and viable tissue as this can give indications for therapeutic targets aimed at reducing hypertrophic scar formation which is initiated by excessive granulation tissue formation.

## 4. Materials and Methods

### 4.1. Human Tissue

Human adult skin with underlying adipose tissue was obtained from healthy individuals undergoing abdominal dermolipectomy. The discarded skin was collected anonymously if patients had not objected to use of their rest material (opt-out system). Eschar was obtained from patients with full-thickness burn wounds undergoing escharotomy. Anonymous tissue collection procedures were performed in compliance with the “Code for Proper Secondary Use of Human Tissue” as formulated by the Dutch Federation of Medical Scientific Societies (www.federa.org) and following procedures approved by the institutional review board of the VU University medical center.

### 4.2. Burn Wound Extract

Eschar was removed 6–21 days post burn from 9 patients with full-thickness burn wounds. Characteristics of the burn wounds and properties of the BWE are shown in Table 1. The upper layers of the eschar were removed and discarded until just above the viable layer. Then the eschar at the interface between non-viable and viable tissue was collected, cut into 0.4 cm^2^ pieces and placed in either 1 mL PBS or 1 mL PBS containing protease inhibitor cocktail (1:100; PIC; Sigma-Aldrich, St. Louis, MO, USA). After two hours gentle shaking at 4 °C the remaining tissue was removed and the solution was centrifuged to pellet any remaining tissue. The supernatant was then filtered using a sterile 0.4 µm pore size filter (Merck Millipore, Amsterdam, the Netherlands) to ensure that all cell and tissue debris was removed as well as any bacteria. This acellular supernatant extracted from the tissue interface between non-viable and viable tissue was collected and stored at −80 °C, and further referred to as BWE. The total protein concentration in the BWE was determined using the Bradford Bio-Rad Protein Assay (BioRad Laboratories, Hercules, CA, USA) as described by the supplier. The BWE contained varying protein concentrations, 2200 ± 1000 µg/mL. For each independent experiment a different cell donor and a BWE isolated from a different donor was used. In the experiments BWE was diluted in the culture medium to 40 and 100 µg/mL to standardize the protein content within each experiment.

### 4.3. Cell Culture

Adipose tissue was carefully dissected from the skin. The remaining skin was then treated with dispase to remove the epidermis from the dermis. The adipose stromal vascular cell fraction and dermal stromal vascular cell fraction were then isolated using collagenase type II/dispase II adipose tissue or dermis as previously described [14].

Dermal fibroblasts (fibroblasts) and ASC were cultured in DMEM (Lonza, Verviers, Belgium), 1% UltroSerG (UG) (BioSepra SA, Cergy-Saint-Christophe, France) and 1% penicillin/streptomycin (P/S) (Invitrogen, Carlsbad, CA, USA).

Endothelial cells were purified from the dermal stromal vascular cell fraction (dermal-EC) and from the adipose stromal vascular cell fraction (adipose-EC) using a MidiMACS separator with microbeads against CD31 as previously described [13]. A >99% pure population (CD31+/CD90−) was obtained at passage 3. The endothelial cells were further cultured on 1% gelatin (Sigma-Aldrich) coated flasks in endothelial cell medium (EC medium): M199 medium (Lonza), 1% P/S, 2 mM l-glutamin (Invitrogen), 10% heat-inactivated New Born Calf Serum (Invitrogen), 10% heat-inactivated Human Serum (Invitrogen), 5 U/mL heparin (Pharmacy VUmc, Amsterdam, The Netherlands) and 3.7 µg/mL endothelial cell growth factor (ECGF; crude extract from bovine brain) (Physiology department VUmc, Amsterdam, The Netherlands).

The cells were stored in the vapor phase of liquid nitrogen until required. For experiments dermal-EC and adipose-EC between passage 5 and 7 were used and fibroblasts and ASC between passage 1 and 3. In all experiments donor-matched cells were used.

### 4.4. Exposure of Endothelial Cells to BWE

Dermal-EC and adipose-EC were seeded in an equal density of 1 × 10^4^ cells/cm^2^ on gelatin-coated culture plates in EC medium. After 16 h the wells were washed twice with HBSS/0.5 mM EDTA before replacing the medium with M199 medium, 10% HS, 10% NBCS, 1% P/S, 2 mM l-glutamin. Monolayers of endothelial cells were exposed to 0, 40 or 100 µg/mL BWE in PIC in HMEC medium for 24 h. Culture supernatants were collected for ELISA.

### 4.5. Cell Migration Assay

Migration of dermal- and adipose-EC was studied as previously described [13]. In short: Dermal- and adipose-EC were seeded in an equal density of 2 × 10^4^ cells/cm^2^ on gelatin-coated culture plates in EC medium. The EC medium was replaced when the cells reached confluency by M199 medium with 10% HS, 10% NBCS, 2 mM l-glutamin and 1% P/S for 24 h before the start of the experiment. A scratch was drawn in a confluent monolayer of dermal- and adipose-EC with a plastic disposable pipette tip. After washing, the cells were exposed to M199 medium with 10% HS, 10% NBCS, 2 mM l-glutamin and 1% P/S supplemented with different concentrations of BWE (0, 40 or 100 µg/mL) or 10 ng/mL bFGF. Phase contrast pictures were taken directly after drawing the scratch and after 16 h of exposure.

Fibroblasts and ASC were seeded in a density of 3.5 × 10^4^ cells/cm^2^ on culture plates in DMEM medium with 1% UltroSerG and 1% P/S [14]. The medium was replaced when the cells reached confluency by DMEM medium with 0.1% Bovine serum albumin and 1% P/S for four days. Then the scratch was drawn through the confluent monolayer with a plastic disposable pipette tip. After washing, the cells were exposed to DMEM medium with 0.1% BSA and 1% P/S supplemented with different concentrations of BWE (0, 40 or 100 µg/mL) or 5 ng/mL EGF. Phase contrast pictures were taken directly after drawing the scratch and 72 h of exposure. Data were analyzed using an image processing algorithm [23]. The closed area was determined by subtracting the open area at time point *t* = 16 h or *t* = 72 h from *t* = 0 h.

### 4.6. Proliferation Assay

Proliferation of dermal- and adipose-EC in response to BWE was determined using ^3^H-thymidine incorporation, adapted from Monsuur et al. [13]. Endothelial cell proliferation was studied in triplicate in low nutrient medium in order to determine their response to BWE. The endothelial cells were seeded on gelatin-coated culture plates in a density of 8 × 10^3^ cells/cm^2^ in M199 medium with 5% HS, 10% NBCS, 2 mM l-glutamin and 1% P/S. Cells were left to adhere to the culture plates for 16 h, followed by 72 h stimulation with either BWE (0, 40, 100 µg/mL in PBS) or bFGF (0, 3 or 10 ng/mL; ReliaTech GmbH, Wolfenbuttel, Germany) or combinations between growth factor and BWE (0, 40, 100 µg/mL). During the last 16 h of growth, 1 µCi ^3^H-thymidine (Perkin Elmer, Belgium) was added to quantify the amount of DNA replication as a measure for proliferation. The β-emission was measured with Ultima Gold scintillation fluid on a Tri-Carb 2800TR Liquid Scintillation Analyzer (PerkinElmer, Zaventem, Belgium).

Proliferation of fibroblasts and ASC in response to BWE was determined in triplicate by manual cell counting. Fibroblasts were seeded on uncoated culture plates in a density of 5 × 10^3^ cells/cm^2^ in DMEM medium with 0.1% Bovine serum albumin and 1% P/S. Cells were left to adhere to the culture plates for 16 h, followed by 56 h stimulation with either BWE (0, 40, 100 µg/mL in PBS) or EGF (0, 10 ng/mL; Sigma-Aldrich). Phase contrast pictures were taken directly after exposure to BWE and after 56 h of exposure. Manual cell counting was performed to determine relative proliferation compared to control.

### 4.7. In Vitro Sprouting Assay

In vitro tube formation was studied using 3D fibrin matrices and dermal- or adipose-EC, as previously described [13]. Briefly, fibrin matrices were prepared by addition of thrombin (0.5 U/mL) (MSD, Haarlem, the Netherlands) to a 3 mg/mL fibrinogen (Enzyme Research Laboratories, Leiden, the Netherlands) solution in M199 medium and 100 µL was added to the wells of a 96-well plate. After polymerization, thrombin was inactivated by incubating the matrices with M199 medium with 10% HS, 10% NBCS, 2 mM l-glutamin and 1% P/S. Dermal- or adipose-EC were seeded to reach a confluent density of 6 × 10^4^ cells/cm^2^. After 16 h, the adipose- and dermal-EC were stimulated with M199 medium with 10% HS, 10% NBCS, 2 mM l-glutamin and 1% P/S and 2 ng/mL TNF-α (ReliaTech GmbH) supplemented with BWE (0, 40, 100 µg/mL in PBS) or bFGF (0, 3 or 10 ng/mL) or BWE and bFGF combined. The sprouts formed by dermal- or adipose-EC into the fibrin matrices were photographed and analyzed using a Nikon Eclipse 80i microscope (Nikon, Tokyo, Japan) and NIS-elements AR software 3.2 (Nikon). The amount of sprouting was measured as percentage surface area of the sprouts of the total surface of the picture.

### 4.8. Histological Staining

Paraffin embedded sections of eschar of 5 µm were stained for morphological analysis (hematoxylin and eosin; HE). The sections were photographed using a Nikon Eclipse 80i microscope (Nikon).

### 4.9. Secretion of Cytokines and Chemokines

ELISAs were performed using commercially available ELISA antibodies. All reagents were used in accordance to the manufacturer’s specifications. IL-6 and CCL2 (both R&D Systems, Abingdon, UK) and CXCL8 (Sanquin, Amsterdam, The Netherlands). ELISA results are expressed in ng/mL.

### 4.10. Statistical Analysis

Statistical analyses were performed using *t*-tests or one-way ANOVA followed by a Dunn’s multiple comparison test. All data was obtained from three to six independent experiments using different cell donors and duplicate wells. The cells in each experiment were donor-matched. Each cell donor was combined with a different BWE donor. Differences were considered significant when * *p* < 0.05, ** *p* < 0.01, *** *p* < 0.001. Results are shown as mean ± SEM.

## Figures and Tables

**Table 1:** Characteristics of burn wounds, properties of the burn wound extract (BWE) and experiments where each BWE donor was used.

#	Gender	Age	Cause of Burn	TBSA	Time after Injury (Days)	Protein Concentration BWE (μg/mL)	BWE Used in Figure
1	female	49	hot water	9%	21	2850	F2
2	female	62	hot object	2%	13	2420	F2
3	female	30	flame	60%	6	1120	F2
4	female	73	flame	2%	17	3260	F4
5	male	49	chemicals	48%	6	720	F3,4,5
6	male	64	hot object	7%	12	1780	F3,4,5
7	male	45	chemicals	0.5%	14	830	F3,4,5
8	female	46	hot object	0.5%	10	1620	F3,4,5
9	male	52	hot water	2.5%	10	2970	F3,4,5

## Supplementary Materials

Supplementary materials can be found at www.mdpi.com/1422-0067/18/8/1790/s1.

## Abbreviations

ASC	Adipose tissue-derived mesenchymal stromal cell
bFGF	Basic fibroblast growth factor
BWE	Burn wound extract
EC	Endothelial cell
EGF	Epidermal growth factor
HE	Hematoxylin and eosin
PIC	Protease inhibitor cocktail
TBSA	Total body surface area
