# Supplementary material for: Burn Eschar Stimulates Fibroblast and Adipose Mesenchymal Stromal Cell Proliferation and Migration but Inhibits Endothelial Cell Sprouting
Source: Int J Mol Sci. 2017 Aug 18;18(8):1790. doi: 10.3390/ijms18081790 (PMC5578178; doi:10.3390/ijms18081790)
Supplement: Supplementary file 1 [file ijms-18-01790-s001.pdf]

Supplementary File

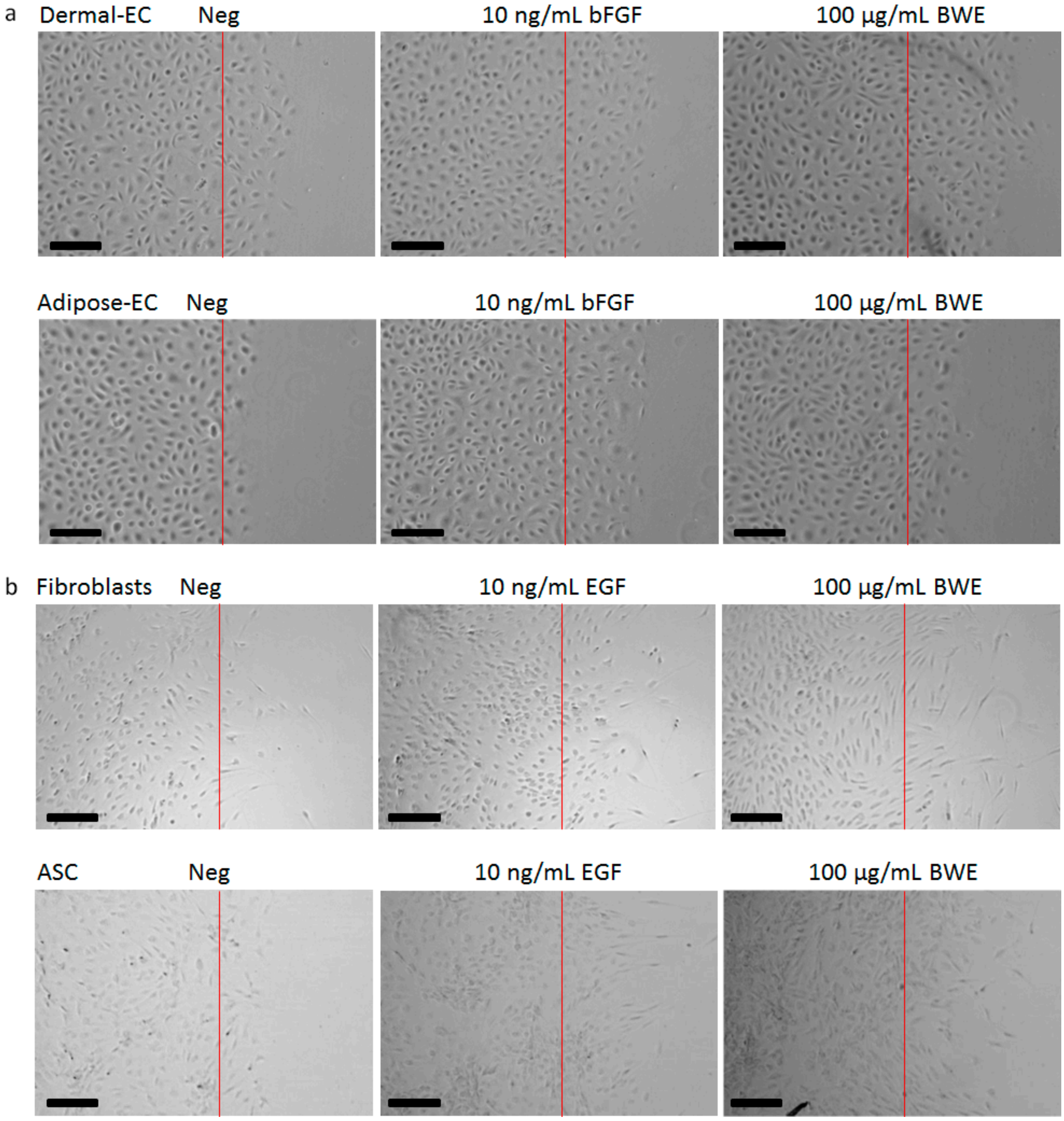

Figure S1. Morphology of dermal- and adipose-endothelial cells, fibroblasts and ASC after exposure to BWE. (**a**) Photographs of migration scratch assay with dermal- and adipose-EC cultured in the presence of 0 (Neg), 10 ng/mL bFGF or 100 μg/mL BWE at t=16 h. (**b**) Photographs of migration scratch assay with fibroblasts and ASC cultured in the presence of 0 (Neg), 10 ng/mL bFGF or 100 μg/mL BWE at t=72 h. Red line indicates the border of the original scratch at t=0 h. Scale bar = 200 μm.
